# Association Between Residence Status Regularization and Access to Healthcare for Undocumented Migrants in Switzerland: A Panel Study

**DOI:** 10.3389/fpubh.2022.832090

**Published:** 2022-05-19

**Authors:** Julien Fakhoury, Claudine Burton-Jeangros, Liala Consoli, Aline Duvoisin, Yves Jackson

**Affiliations:** ^1^Swiss National Center of Competence in Research (NCCR) “LIVES – Overcoming Vulnerability: Life Course Perspectives”, University of Geneva, Geneva, Switzerland; ^2^Institute of Sociological Research, University of Geneva, Geneva, Switzerland; ^3^Center for the Interdisciplinary Study of Gerontology and Vulnerability, University of Geneva, Geneva, Switzerland; ^4^Geneva University Hospital and University of Geneva, Geneva, Switzerland

**Keywords:** undocumented migrants, healthcare utilization, access to healthcare, policy, residence status regularization

## Abstract

**Background:**

Switzerland has a universal healthcare system. Yet, undocumented migrants face barriers at different levels that hinder their access to healthcare services. The aim of this study is to assess whether undocumented migrants' healthcare utilization improves with residence status regularization.

**Methods:**

We used two-wave panel data from the Parchemins study, a study exploring the impact of regularization on undocumented migrants' health in Geneva, Switzerland. First wave data were collected between 2017 and 2018, second wave data between 2019 and 2020. At baseline, the sample consisted of 309 undocumented migrants, recruited after the implementation of a temporary regularization policy in Geneva. We distributed them into two groups according to their residence status 12 months before the second data collection [regularized vs. undocumented (controls)]. Using as dependent variable the number of medical consultations within two distinct 12-months periods (the first before regularization, the second after regularization), we conducted multivariable regression analyses applying hurdle specification to identify factors enhancing healthcare utilization. Then, we estimated first-difference panel models to assess change in healthcare utilization along regularization. Models were adjusted for demographic, economic and health-related factors.

**Results:**

Of the 309 participants, 68 (22%) were regularized. For the 12 months before regularization, these migrants did not significantly differ in their healthcare utilization from the controls. At this stage, factors increasing the odds of having consulted at least once included being a female (aOR: 2.70; 95% CI: 1.37–5.30) and having access to a general practitioner (aOR: 3.15; 95% CI: 1.62–6.13). The factors associated with the number of consultations apart from underlying health conditions were the equivalent disposable income (aIRR per additional CHF 100.-: 0.98; 95% CI: 0.97–1.00) and having access to a general practitioner (aIRR: 1.45; 95% CI: 1.09–1.92). For the 12 months after regularization, being regularized was not associated with higher odds of having consulted at least once. However, among participants who consulted at least once, regularized ones reported higher counts of medical consultations than controls (3.7 vs. 2.6, *p* = 0.02), suggesting a positive impact of regularization. Results from the first-difference panel models confirmed that residence status regularization might have driven migrants' healthcare utilization (aβ: 0.90; 95% CI: 0.31-1.77).

**Conclusions:**

This study supports the hypothesis that residence status regularization is associated with improved healthcare utilization among undocumented migrants. Future research is needed to understand the mechanisms through which regularization improves undocumented migrants' use of healthcare services.

## Background

Over the past few years, the International Community reaffirmed on multiple occasions its commitment toward universal health coverage (UHC), defined by the World Health Organization as ensuring the right of everyone to access quality healthcare in times of need and at an affordable price (1–3). Notably, UHC was framed as one of the main millennium and sustainable development goals adopted by the United Nations (1–3). Yet, undocumented migrants, i.e. migrants settled in a host country without a valid residence authorization, often face legal restrictions that limit their access to healthcare. For instance, in Europe, only few countries provide undocumented migrants with an access to regular primary and secondary healthcare (4). Most restrict undocumented migrants' entitlement to emergency services, sometimes at a substantial cost that prevents them from seeking care (4).

These structural (legal, administrative) restrictions cumulate with barriers to healthcare at the individual level. The Behavioral model, a theoretical framework specifically developed to explain healthcare access and utilization, differentiates the predisposing from the enabling factors. The predisposing factors refer to sociodemographic characteristics such as age, gender, ethnicity, level of acculturation or immigration status. The enabling factors encompass the persons' resources to access healthcare and cover its costs (5, 6). In the case of undocumented migrants, factors linked to their lack of residence status such as fear of denunciation, social marginalization, stigmatization or limited financial resources have consistently been found to hinder their access to healthcare (5, 7–11). For instance, in Switzerland, Germany and Denmark, undocumented pregnant women tended to avoid or delay pre-natal consultations due to lack of knowledge about the healthcare system, socioeconomic hardships or fear of being identified as undocumented (12–14). Undocumented migrants in Denmark also reported poor language proficiency and lack of social relationships with Danish people as major barriers to healthcare (9). These barriers are not specific to the European context but also exist in other parts of the world, such as in South Asia. For example, a study in India found that undocumented Bangladeshi and Nepalese migrants did not frequently use the local healthcare systems due to social exclusion and fear of identification or discrimination (15). Overall, compared to regular migrants, studies conducted in several European countries showed that undocumented migrants were less likely to seek healthcare (8, 12, 16).

On the other hand, undocumented migrants cumulate risk factors for poor health that may increase their needs for care. They show a high prevalence of multiple chronic conditions, which has been found to be a key determinant of their healthcare demand (17). They frequently occupy physically demanding jobs and are exposed to abuses on the labor market and to workplace violations (18–21). They have consistently been shown to be at higher risk of infections such as sexually transmitted diseases (22–25). Their often limited life opportunities and social interactions increase their risk of suffering from psychological distress (26). Overall, studies conducted across Europe showed that undocumented migrants consistently reported poorer physical and mental health than regular migrants or native residents (27–29).

This discrepancy between undocumented migrants' health needs and their effective use of the healthcare system may contribute to the development of serious yet preventable health problems among this population. In turn, this could lead to higher medical costs borne by healthcare systems and society as a whole (30). Designing efficient policies to bridge this gap is therefore of crucial medical, public health and economic importance (31, 32). A study among undocumented women in Utah, United States (US), suggested that public policies aiming at favoring undocumented migrants' social integration increased their healthcare utilization (33). In California, the enrolment of undocumented children in health insurance plans improved their access to and their use of medical and dental care (34). In view of this evidence, one may expect inclusive policies such as residence status regularization to contribute to the alleviation of structural and individual barriers to healthcare for undocumented migrants, resulting in improved healthcare access and increased utilization. However, evidence regarding the impact of residence status change on undocumented migrants' healthcare utilization is scarce. In Europe, to the best of our knowledge, no study has addressed the association between residence status regularization and use of healthcare services. This article attempts to fill this gap using longitudinal data from the Parchemins Study, a study evaluating the impact of the residence status regularization on undocumented migrants' living conditions and health in Geneva, Switzerland. More specifically, this paper tests whether residence status regularization leads to increased healthcare utilization among undocumented migrants.

## Methods

### Setting

According to the latest estimates, the Canton of Geneva (population 500,000 inhabitants), Switzerland, is home to 10,000–15,000 undocumented migrants (35). Most of these migrants are well-established workers who lack valid residence authorization (undocumented economic migrants). Failed asylum seekers account for a small share (36). The healthcare system in Switzerland is universal but requires the individual purchase of a mandatory private insurance. Against payment of a premium of CHF 375—(344 €/405 USD) per month on average, this insurance covers a wide range of preventive, curative as well as rehabilitation services, provided that the patient has first paid a fixed deductible ranging from CHF 300—(250 €/324 USD) to CHF 2,500—(2,300 €/2,698 USD) entirely out of pocket. According to the Swiss legislation, undocumented migrants are entitled to and obliged, like any other resident, to take out a private health insurance upon 3 months of residence in the country. However, compliance with this obligation is only verified for individuals with a valid residence authorization.

In the Canton of Geneva, only 13–16% undocumented migrants are effectively insured (16% in our sample) (26, 37). Barriers faced by undocumented migrants to the purchase of an insurance include fear of denunciation, limited awareness of their rights or insufficient economic resources (36). In order to broaden healthcare access and utilization for undocumented and uninsured population, the Geneva University Hospital (HUG) dedicated a health center that provides a comprehensive range of subsidized medical services. Free medical consultations are also organized in the community, where general practitioners serve voluntarily as family doctors for undocumented migrants.

In 2017–2018, the Canton of Geneva implemented a two-year pilot policy called “Operation Papyrus”. Its aim was to grant undocumented economic migrants renewable residence authorizations upon the following strict requirements: (1) no previous application for asylum, (2) a continuous stay in Geneva for 10 years (5 years for parents of school-aged children), (3) financial independence, (4) basic French proficiency, and (5) absence of criminal record. Meeting these criteria, jointly agreed upon by the local authorities, trade unions and non-governmental associations, guaranteed migrants who applied for regularization to be granted a residence authorization. Policy implementation also involved trade unions and non-governmental associations (NGO's) with a mandate from the local authorities (1) to act as gatekeepers and (2) to assist eligible migrants throughout the regularization process.

After regularization, migrants had 3 months to enroll into a health insurance scheme. Once insured, they could no longer consult at the HUG dedicated unit for undocumented and uninsured population but could access to the whole range of services within the regular healthcare system. In this context, two hypotheses are tested here. On the one hand, regularization is expected to increase the uptake of the mandatory health insurance scheme, resulting in improved healthcare utilization among regularized migrants. On the other hand, regularized migrants could face difficulties in meeting the healthcare costs generated by the Swiss mandatory health insurance while also losing access to the HUG dedicated unit for uninsured population, which in turn could hamper their healthcare utilization (38).

### Study Design

This is a longitudinal, observational study based on two-wave data collected within the larger framework of the Parchemins Study. The Parchemins Study protocol can be consulted elsewhere (38).

### Participants

Participants were recruited in Geneva between October 2017 and December 2018. At baseline, the sampled population consisted of undocumented economic migrants who (1) were aged 18 or more, (2) were not nationals of a European Union or European Free-Trade Association member state, (3) had never been asylum seekers, and (4) had been residing continuously in Geneva for at least 3 years. It included migrants who had been regularized within 3 months prior to their participation, a timeframe deemed too short to allow significant shifts due to regularization in their living conditions.

Recruitment strategies were set up in order to ensure a convenience sample as diverse as possible, taking into account that undocumented economic migrants are hard-to-reach. The main strategy consisted of face-to-face recruitment at two different settings: (1) during sessions organized by mandated trade unions and NGO's to assist undocumented economic migrants with their application for regularization (85%) and (2) in the waiting room of the HUG dedicated unit for undocumented and uninsured population (15%). Secondary strategies included snowball sampling and advertising through social networks.

Before they participated in the first data collection, all participants were ensured confidentiality orally and in writing and were asked to fill in an informed consent form. Those who consented to participate in the second data collection were asked for their phone numbers and e-mail addresses, so that we could recontact them approximately 12 months later. We then prioritized phone contact for the follow-up strategy and resorted to e-mail messages in case of non-response (38).

### Data Collection

Data were collected face-to-face by trained investigators, using a mobile tablet with a pre-loaded questionnaire (Computer-Assisted Personal Interviews). The questionnaire consisted of measurements of variables related to participants' (1) sociodemographic characteristics and residence status, (2) living conditions, (3) health and access to healthcare, (4) economic and financial situation, and (5) social relationships. It was specifically developed for the purpose of longitudinal data collection in the context of the Parchemins Study and was used for the first and second data collections, with slight adjustments before the latter. The use of the same questionnaire for both data collections allowed for comparisons over time of the same variables measured at different time points.

The questionnaire was translated into the four main languages spoken by undocumented economic migrants in Geneva (French, English, Portuguese and Spanish). It was completed at the University of Geneva or at a place chosen by the participants, in their preferred language. The participants entered their responses in the mobile tablet with the assistance of the investigators. First wave data were gathered between October 2017 and December 2018 and second wave data between March 2019 and February 2020. On average, the time elapsed between the first and second personal interviews was 15 months.

The Ethics Committee of the Geneva Canton, Switzerland, approved the study protocol (CCER 2017-00897).

### Variables

#### Measure of Healthcare Utilization

Healthcare utilization was measured using a discrete variable, the self-reported number of consultations to a medical doctor in the previous 12 months, which is an indicator widely used in the literature (39–42). The number of consultations ranged from 0 to 10+ (10+ meaning “10 consultations or more”). Medical consultations encompassed visits to a general practitioner, specialist, psychiatrist or gynecologist, but excluded consultations with a dentist, which are not covered by health insurance in Switzerland, and visits to emergency rooms. Given that participants in the regularized group had obtained a residence permit at most for 3 months at the moment of the first data collection, we assumed that the number of consultations reported for the 12 months prior to the first personal interviews reflected participants' healthcare utilization while undocumented.

#### Residence Status

**Figure 1:**
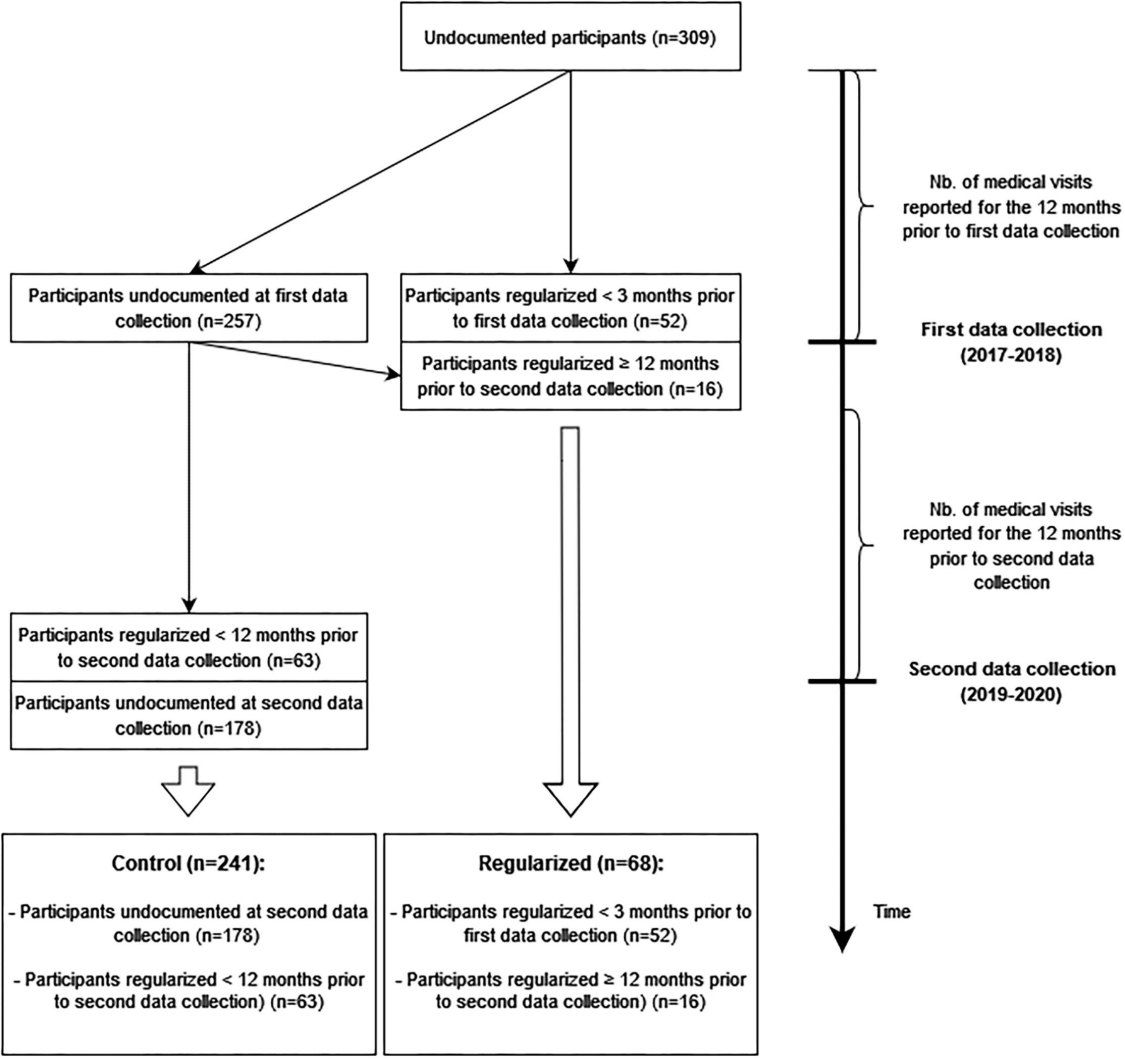
Assignment of participants to residence status groups over time.

Residence status regularization was our main exposure of interest. We categorized participants into two groups, based on the evolution of their residence status between the two data collection periods (Figure 1). The regularized group encompassed migrants that had been regularized 12 months or more prior to their second wave personal interviews. This categorization allowed us to explore within-individual covariance over time between healthcare utilization and residence status regularization. Indeed, we assumed that over the 12 months prior to their second wave personal interviews, these regularized migrants had insurance and faced fewer constraints on seeking healthcare as compared to when they were undocumented. Alternatively, the control group included (1) migrants who remained undocumented at the time of the second data collection and (2) migrants who got a residence authorization <12 months prior to their second wave personal interviews. We merged this latter subgroup with the undocumented participants to limit temporality bias, since we could not determine whether the medical visits that they reported occurred prior to their regularization—that is, if they occurred despite facing barriers to healthcare related to their lack of legal status—or after they had been regularized.

#### Covariates

Covariates were selected following the Behavioral model (5) and taking into account the eligibility criteria for regularization in the context of the Operation Papyrus.

As predisposing covariates, we included age, sex, country of origin (Latin America as reference, Asia, Eastern Europe, Africa), the duration of stay in Geneva (in years) and the level of oral French proficiency (Good as reference, Fair, Poor). These two latter variables were specifically included to reduce the risk of confounding bias, since they could predict residence status regularization in the context of the Operation Papyrus.

We used the equivalent disposable income [per CHF 100—units (95€/100 USD)], which was also a regularization criterion, the transfer of remittances (Yes vs. No) as well as having access to a general practitioner (GP) (Yes vs. No) as measures of enabling factors. Sending remittances was included as a complementary measure of the financial resources. Specifically, since remittances are often budgeted for relatives living in the country of origin or abroad, sending remittances might reduce participants' financial resources available in the event of healthcare needs. Having access to a GP was measured asking participants if they had a doctor to whom they could go for most of their health problems, regardless of whether the doctor was employed at the HUG or operating elsewhere. Despite a hypothesized mediating effect, we did not include health insurance as an enabling covariate in our analyses due to collinearity issues.

Health needs factors were measured using (1) the presence of comorbidity, defined as the self-report of 3 or more somatic chronic conditions (43) and (2) the self-report of depression or anxiety. The chronic conditions used to define comorbidity were selected in accordance with the Swiss Health Survey (SHS) (see 2.5.4 Secondary data source for a description of SHS). They included: asthma, any chronic lung disease, any heart disease, hypertension, a stroke, chronic joint pain, chronic back pain, chronic neck pain, diabetes, cholesterol, osteoporosis, any allergy, any liver disease and any kidney disease (44).

Age, the enabling factors and the variables related to the health needs were measured at baseline and at follow-up. Sex, origin, the duration of stay in Geneva and the level of oral proficiency in French were only measured at baseline.

#### Secondary Data Source

To compare participants' healthcare utilization with the general population's use, a secondary random sample of 580 natives and legal residents in Geneva, comparable in terms of age range and occupational status, was drawn from the 2017 Swiss Health Survey (SHS). The SHS is a survey held every 5 years whose aim is to describe the health status as well as the healthcare consumption of the Swiss population (44).

### Statistical Analyses

Categorical variables are presented as absolute numbers and relative percentages. Continuous variables are presented as means and standard deviations (SD). Cross-sectional comparisons across residence status groups were made using the Chi-square test or the Mann-Whitney's U-test, as appropriate. Changes over time in the outcome, the enabling and the health needs factors were measured using the McNemar's Chi-square test or the Wilcoxon's Signed-Rank Test, as appropriate. Statistical significance was set at 0.05.

First, we ran bivariate analyses to compare participants' healthcare utilization at baseline with that of the general population in Geneva. The comparison was not adjusted for predisposing and enabling covariates, since the 2017 SHS did not include fully comparable measures.

In a second step, we conducted univariate and multivariate count regression analyses to identify the factors associated with healthcare utilization among participants for the two 12-months periods studied; first, for the 12 months prior to the first data collection and second, for the 12 months before the second data collection. For the period prior to the first data collection, we were particularly interested in determining whether regularized migrants already differed from the controls in terms of medical visits. For the 12 months before the second data collection, we specifically looked for an association between regularization and healthcare utilization.

We used hurdle models to account for zero-inflation and over-dispersion in the outcome. Hurdle models also allowed emphasizing two distinct processes underlying healthcare utilization. The first process distinguished users from non-users of healthcare services, i.e., modeled the odds of reporting at least one medical visit, using logit regressions (the hurdle parts). Results of the hurdle parts are presented as odds ratio (OR) and 95% confidence intervals (95% CI) for univariate regressions and as adjusted odds ratio (aOR) and 95% CI for multivariate regressions. The second process assessed the factors associated with higher counts of medical consultations among healthcare users using truncated-at-zero negative binomial regressions (the truncated parts). Results of the truncated parts are presented as incidence risk ratios (IRR) and 95% CI for univariate regressions and as adjusted incidence risk ratios (aIRR) and 95% CI for multivariate regressions.

In a third step, we estimated panel models using the first-difference estimator to assess change in healthcare utilization associated with regularization. The first-difference estimator controlled for time-invariant unobserved heterogeneity and thus allowed for the exploration of within-individual covariance over time. Results of the first-difference panel models are presented as adjusted beta coefficients (aβ) and 95% CI. All the analyses were run using R (version 4.0).

## Results

### Sample Description

This study included 309 participants, predominantly women (72.8%) originating from Latin America (64.7%) or East Asia (22%) (Table 1). Of these 309 participants, 68 (22%) belonged to the regularized group. At baseline, the mean age of the participants was 43.9 years (SD: 10.3). Regardless of the residence status, most participants reported at least a fair level of French proficiency (80.5%) and the mean duration of stay in Geneva was 11.9 years (SD: 4.8). Nonetheless, participants in the regularized group reported better French proficiency and had resided significantly longer in Geneva than those in the undocumented group.

**Table 1:** Sociodemographic characteristics of the study participants, stratified by residence status (*N* = 309).

	**Total (*N =* 309)**	**Control group (*N =* 241)**	**Regularized group (*N =* 68)**	***p*** **-value**
Female	225 (72.8%)	171 (71%)	54 (79.4%)	0.166
Age^a^	43.9 (10.3)	43.5 (10.5)	45.7 (9.1)	0.086
Origin				0.048
Latin America	200 (64.7%)	146 (60%)	54 (79.4%)	
Africa	17 (5.50%)	15 (6.2%)	2 (2.9%)	
East Asia	68 (22%)	59 (24.5%)	9 (13.2%)	
Eastern Europe	24 (7.8%)	21 (8.7%)	3 (4.4%)	
Duration in Geneva^a^	11.9 (4.8)	11.3 (5%)	13.7 (3.8)	<0.001
Oral French proficiency				0.005
Good	133 (43%)	97 (40.2%)	36 (52.9%)	
Fair	116 (37.5%)	88 (36.5%)	28 (41.2%)	
Poor	60 (19.5%)	56 (23.2%)	4 (5.9%)	

a *Presented as mean (SD)*.

At baseline, the mean equivalent disposable income was CHF 2348—(2157 €; 2539 USD) [SD: CHF 1159—(1064 €; 1253 USD)] the proportion of participants sending remittances to their home country was 69.6% and the proportion of participants having access to a GP was 35% (Table 2). The mean equivalent disposable income remained stable over time among regularized participants, while it slightly but significantly increased among undocumented ones. The proportion of participants sending remittances significantly decreased over time in the regularized group, but remained stable overall. In both groups, the number of participants who reported having access to a GP significantly increased.

**Table 2:** Levels of enabling and health needs factors at each period, stratified by residence status.

	**Total (*N* = 309)**		***p*** **-value**	**Control group (*N* = 241)**		***p*** **-value**	**Regularized group (*N* = 68)**		***p*** **-value**
	**Wave 1**	**Wave 2**		**Wave 1**	**Wave 2**		**Wave 1**	**Wave 2**	
Equivalent disposable income (in CHF.-)	2,348 (1,159)	2,441 (1,075)	0.031	2,205 (1,209)	2,346 (1098)	0.018	2,854 (777)	2,777 (918)	0.872
Transfer of remittances	215 (69.6%)	210 (68%)	0.508	170 (70.5%)	173 (71.8%)	0.647	45 (66%)	37 (54.4%)	0.033
Having access to a GP	108 (35%)	153 (49.5%)	<0.001	76 (31.5%)	97 (40.2%)	0.006	32 (47.1%)	56 (82.4%)	<0.001
Comorbidity	51 (16.5%)	73 (23.6%)	0.003	42 (17.4%)	62 (25.7%)	0.004	9 (13.2%)	11 (16.2%)	0.727
Depression or anxiety	57 (18.4%)	62 (20.1%)	0.484	50 (20.7%)	59 (24.5%)	0.170	7 (10.3%)	3 (4.4%)	0.289

While the proportion of participants suffering from comorbidity significantly rose from 16.5% (Wave 1) to 23.6% (Wave 2), the proportion of participants reporting depression or anxiety did not significantly change over time.

### Medical Consultations in the 12 Months Before the First Data Collection

**Figure 2:**
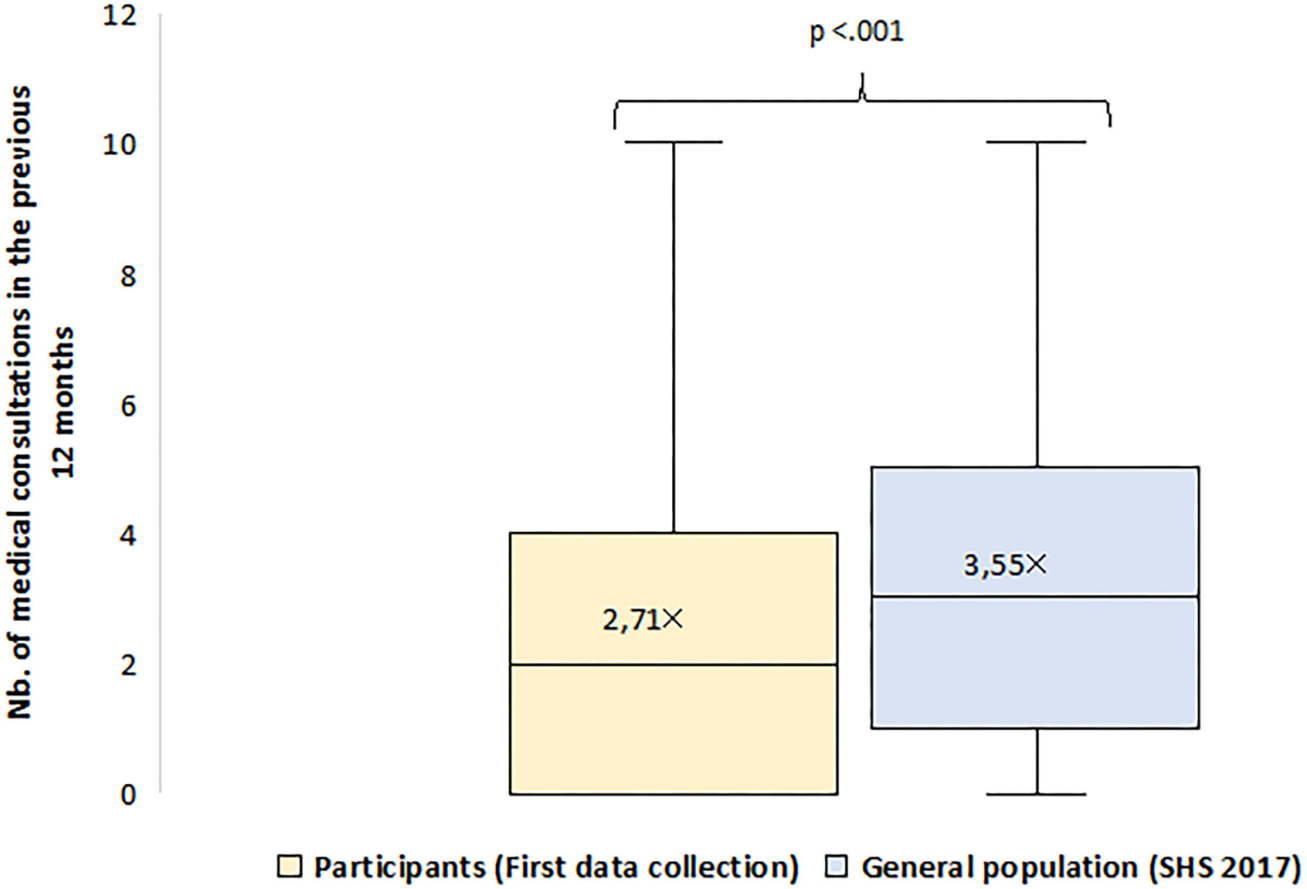
Healthcare utilization of study participants (first data collection), as compared to the general population in Geneva.

Figure 2 displays participants' self-reported number of medical consultations in the 12 months prior to the first data collection, as compared to the estimates for 2017 for the general population in Geneva. While all undocumented, participants reported significantly fewer consultations than the general population in Geneva, with the lower quartiles taking on the values of 0 and 1 respectively, the medians of 2 and 3 and the upper quartiles of 4 and 5 respectively. On average, participants reported 2.7 consultations compared to 3.6 for the general population.

Either in the univariate (OR: 0.91; 95% CI: 0.50–1.67) [Table 3, Hurdle part (1)] or multivariate analyses (aOR 0.88; 95% CI: 0.44–1.77) [Table 4, Hurdle part (1)], regularized participants were not more likely to have had at least one medical consultation in the last 12 months than controls. In the multivariate analysis, only being a female (aOR: 2.70; 95% CI: 1.37–5.30), having access to a GP (aOR: 3.15; 95% CI: 1.62–6.13) and suffering from comorbidity (aOR: 6.01; 95% CI: 1.73–20.84) significantly increased the odds of having at least one medical consultation in the last 12 months.

**Table 3:** Univariate associations between the number of consultations in the previous 12 months and predisposing, enabling and health needs factors.

	**Consultations in the previous 12 months prior to the first wave personal interviews**				**Consultations in the previous 12 months prior to the second wave personal interviews**			
	**Hurdle part (1)** **(*N* = 309)**		**Truncated part (1)** **(*N* = 227)**		**Hurdle part (2)** **(*N* = 309)**		**Truncated part (2)** **(*N* = 218)**	
	**OR (95% CI)**	***p*** **-value**	**IRR (95% CI)**	***p*** **-value**	**OR (95% CI)**	***p*** **-value**	**IRR (95% CI)**	***p*** **-value**
Regularized (ref. Controls)	0.91 (0.50, 1.67)	0.767	1.05 (0.72, 1.53)	0.781	1.21 (0.66, 2.21)	0.542	1.55 (1.09, 2.22)	0.016
Female (ref. Male)	2.92 (1.70, 5.01)	<0.001	1.05 (0.72, 1.54)	0.805	2.68 (1.58, 4.55)	<0.001	1.19 (0.80, 1.76)	0.390
Age (per additional year)	1.00 (0.98, 1.03)	0.958	1.00 (0.98, 1.01)	0.629	1.00 (0.98, 1.03)	0.725	1.00 (0.99, 1.02)	0.460
Origin: Asia (ref. Latin America)	0.50 (0.27, 0.93)	0.028	0.71 (0.48, 1.06)	0.095	0.71 (0.38, 1.30)	0.263	1.03 (0.70, 1.52)	0.869
Origin: Eastern Europe (ref. Latin America)	0.26 (0.11, 0.62)	0.002	1.29 (0.67, 2.48)	0.451	0.19 (0.08, 0.46)	<0.001	1.58 (0.74, 3.37)	0.239
Origin: Africa (ref. Latin America)	0.47 (0.17, 1.35)	0.163	1.60 (0.82, 3.12)	0.167	0.45 (0.16, 1.25)	0.126	1.63 (0.79, 3.36)	0.182
Oral proficiency in French: Fair (ref. Good)	1.15 (0.65, 2.01)	0.632	0.94 (0.66, 1.33)	0.713	1.28 (0.73, 2.23)	0.387	0.84 (0.60, 1.20)	0.342
Oral proficiency in French: Poor (ref. Good)	1.31 (0.65, 2.66)	0.448	0.95 (0.62, 1.44)	0.796	0.89 (0.46, 1.71)	0.729	0.78 (0.50, 1.22)	0.272
Duration in Geneva (per additional year)	0.98 (0.93, 1.03)	0.346	1.01 (0.98, 1.05)	0.416	0.98 (0.93, 1.04)	0.558	1.03 (0.99, 1.06)	0.107
Equivalent disposable income (per additional CHF 100.-)	0.97 (0.95, 0.99)	0.008	0.99 (0.97, 1.00)	0.035	0.94 (0.92, 0.97)	<0.001	0.99 (0.98, 1.01)	0.230
Transfer of remittances (ref. No transfer)	0.73 (0.41, 1.29)	0.270	0.77 (0.56, 1.07)	0.114	0.79 (0.47, 1.36)	0.399	0.87 (0.63, 1.22)	0.431
Having access to a GP (ref. No access to GP)	2.56 (1.41, 4.64)	0.002	1.28 (0.94, 1.75)	0.119	1.66 (1.01, 2.72)	0.045	2.05 (1.53, 2.76)	<0.001
Comorbidity (ref. Absence of comorbidity	7.06 (2.14, 23.35)	0.001	1.73 (1.22, 2.45)	0.002	2.89 (1.44, 5.79)	0.003	1.54 (1.11, 2.16)	0.011
Depression or anxiety (ref. Absence of depression and anxiety)	1.88 (0.90, 3.92)	0.092	1.88 (1.33, 2.65)	<0.001	1.55 (0.80, 2.98)	0.208	1.27 (0.87, 1.85)	0.187

**Table 4:** Multivariate associations between the number of consultations in the previous 12 months and predisposing, enabling and health needs factors.

	**Consultations in the previous 12 months prior to the first wave personal interviews**				**Consultations in the previous 12 months prior to the second wave personal interviews**			
	**Hurdle part (1)** **(*N* = 309)**		**Truncated part (1)** **(*N* = 227)**		**Hurdle part (2)** **(*N* = 309)**		**Truncated part (2)** **(*N* = 218)**	
	**aOR (95% CI)**	***p*** **-value**	**aIRR (95% CI)**	***p*** **-value**	**aOR (95% CI)**	***p*** **-value**	**aIRR (95% CI)**	***p*** **-value**
Regularized (ref. Controls)	0.88 (0.44, 1.77)	0.716	1.18 (0.83, 1.66)	0.353	0.96 (0.46, 2.01)	0.904	1.50 (1.07, 2.09)	0.018
Female (ref. Male)	2.70 (1.37, 5.30)	0.004	1.21 (0.82, 1.79)	0.335	2.31 (1.20, 4.44)	0.012	1.32 (0.91, 1.91)	0.141
Age (per additional year)	0.99 (0.96, 1.02)	0.578	0.99 (0.97, 1.00)	0.135	0.99 (0.95, 1.02)	0.380	0.99 (0.98, 1.01)	0.369
Origin: Asia (ref. Latin America)	0.53 (0.26, 1.09)	0.083	0.74 (0.50, 1.08)	0.114	0.93 (0.45, 1.92)	0.835	1.09 (0.81, 1.56)	0.621
Origin: Eastern Europe (ref. Latin America)	0.62 (0.21, 1.82)	0.384	2.07 (1.06, 4.02)	0.032	0.40 (0.13, 1.18)	0.096	1.41 (0.70. 2.83)	0.335
Origin: Africa (ref. Latin America)	0.53 (0.16, 1.76)	0.302	1.32 (0.71, 2.46)	0.374	0.34 (0.10, 1.13)	0.077	1.97 (1.07, 3.64)	0.029
Oral proficiency in French: Fair (ref. Good)	1.05 (0.54, 2.04)	0.878	1.08 (0.80, 1.48)	0.607	1.01 (0.52, 1.96)	0.972	0.94 (0.69. 1.27)	0.671
Oral proficiency in French: Poor (ref. Good)	1.17 (0.47, 2.92)	0.730	1.07 (0.71, 1.60)	0.760	0.56 (0.24, 1.33)	0.191	0.95 (0.63, 1.45)	0.820
Duration in Geneva (per additional year)	0.96 (0.89, 1.03)	0.272	1.02 (0.99, 1.06)	0.246	0.98 (0.92, 1.05)	0.570	1.01 (0.97, 1.04)	0.717
Equivalent disposable income (per additional CHF 100.-)	0.98 (0.95, 1.00)	0.091	0.98 (0.97, 1.00)	0.019	0.94 (0.91, 0.97)	<0.001	0.98 (0.96, 0.99)	0.004
Transfer of remittances (ref. No transfer)	0.78 (0.40, 1.53)	0.470	0.93 (0.69, 1.26)	0.648	1.35 (0.69, 2.61)	0.382	1.22 (0.90, 1.65)	0.191
Having access to a GP (ref. No access to GP)	3.15 (1.62, 6.13)	<0.001	1.45 (1.09, 1.92)	0.011	2.43 (1.28, 4.61)	0.006	2.30 (1.70, 3.11)	<0.001
Comorbidity (ref. Absence of comorbidity)	6.01 (1.73, 20.84)	0.005	1.46 (1.06, 2.00)	0.019	2.61 (1.21, 5.65)	0.015	1.42 (1.03, 1.94)	0.027
Depression or anxiety (ref. Absence of depression and anxiety)	1.29 (0.56, 2.97)	0.547	1.80 (1.29, 2.51)	<0.001	0.84 (0.39, 1.83)	0.667	1.22 (0.87, 1.72)	0.251

Among participants who reported at least one medical consultation, regularized and control participants reported comparable counts of consultations (aIRR: 1.18; 95% CI: 0.83–1.66) [Table 4, Truncated part (1)]. In both univariate and multivariate analyses, having access to a GP (aIRR: 1.45; 95% CI: 1.09–1.92), suffering from comorbidity (aIRR: 1.46; 95% CI: 1.06–2.00) and reporting depression or anxiety (aIRR: 1.80; 95% CI: 1.29–2.51) were the only factors associated with more medical consultations. On the other hand, a higher equivalent disposable income was negatively associated with visits counts (aIRR per additional CHF 100.-: 0.98; 95% CI: 0.97–1.00).

### Medical Consultations in the 12 Months Before the Second Data Collection

**Figure 3:**
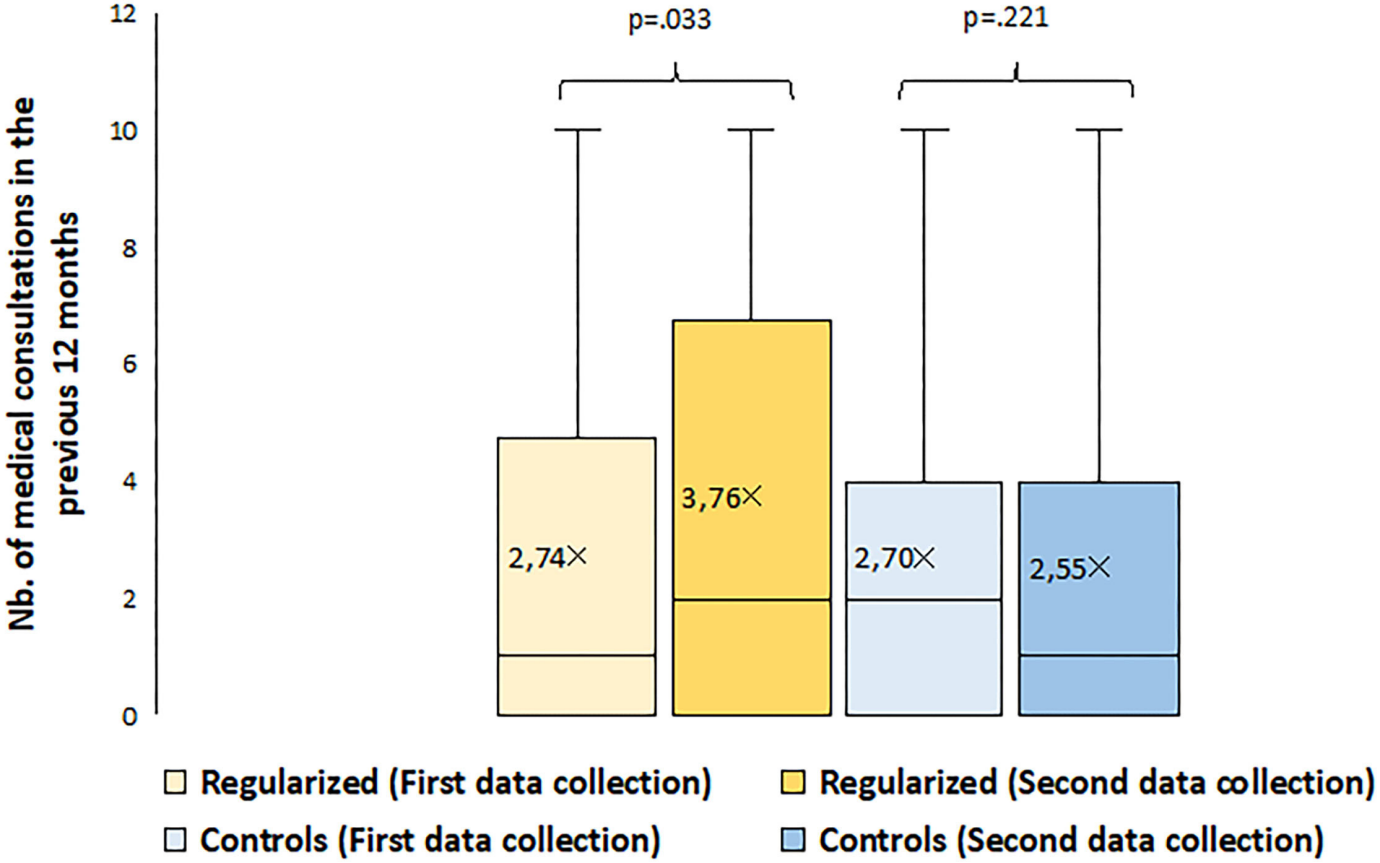
Evolution of participants' healthcare utilization, stratified by residence status groups.

While the number of medical consultations significantly increased between the first and second data collections among regularized participants, it remained stable in the control group, suggesting a positive relationship between regularization and healthcare utilization (Figure 3). Specifically, the average number of reported medical consultations significantly rose from 2.7 to 3.7 among the regularized group, while it non-significantly decreased from 2.7 to 2.6 in the control group.

Overall, factors associated with increased odds of having visited a medical doctor in the 12 months prior to the second data collection were consistent with the results of the first data collection. Specifically, participants in the control group were still as likely as regularized ones to have consulted at least once (aOR: 0.96; 95% CI: 0.46–2.01) In the multivariate analysis, being a female (aOR: 2.31; 95% CI: 1.20-4.44), having access to a GP (aOR: 2.43; 95% CI: 1.28-4.61), comorbidity (aOR: 2.61; 95% CI: 1.21-5.65) and a lower equivalent disposable income (aOR per additional CHF 100.-: 0.94; 95% CI: 0.91-0.97) significantly increased the odds of reporting at least one consultation.

However, among participants who visited a doctor at least once, regularized ones reported significantly higher counts of medical consultations than controls (IRR: 1.55; 95% CI: 1.09–2.22) [Table 3, Truncated part (2)]. This difference remained significant after adjustment for predisposing, enabling and health needs factors (aIRR: 1.50; 95% CI: 1.07, 2.09) [Table 4, Truncated part (2)]. A lower equivalent disposable income (aIRR per additional CHF 100.-: 0.98; 95% CI: 0.96–0.99), having access to a GP (aIRR: 2.30; 95% CI: 1.70–3.11) and suffering from comorbidity (aIRR: 1.42; 95% CI: 1.03–1.94) also remained significantly associated with higher counts of medical consultations.

### Within-Individual Covariance Between Healthcare Utilization and Residence Status Regularization

Results from the first-difference panel model adjusted for time-varying enabling and health needs factors provided further support for a positive association at the individual level between residence status regularization and healthcare utilization. Indeed, regularization of residence status was associated with an increase in the number of medical consultations (aβ: 0.90; 95% CI: 0.31–1.77) (Table 5). The equivalent disposable income (aβ per additional CHF 100.-: −0.04; 95% CI: −0.07–0.00) as well as having access to a GP (aβ: 0.86; 95% CI: 0.15–1.56) were also significant predictors of change in the number of medical visits.

**Table 5:** Within-individual variation in healthcare utilization according to residence status regularization, enabling and health needs factors.

	**Change in the number of medical consultations**	
	**Beta coefficients (95% CI)**	***p*** **-value**
Regularization (> 12 months prior to the second wave personal interview) (ref. Undocumented or regularization < 12 months)	0.90 (0.31, 1.77)	0.043
Equivalent disposable income (per additional CHF 100.-)	−0.04 (−0.07, 0.00)	0.043
Transfer of remittances (ref. No transfer)	0.12 (−0.71, 0.94)	0.781
Having access to a GP (ref. No access to GP)	0.86 (0.15, 1.56)	0.017
Comorbidity (ref. Absence of comorbidity)	−0.15 (−0.98, 0.68)	0.723
Depression or anxiety (ref. Absence of depression and anxiety)	0.23 (−0.63, 1.09)	0.602
F-statistic	2.90	0.009
Within R-squared	0.05	

## Discussion

Using two-wave panel data, this study provides evidence of a positive association between healthcare utilization and residence status regularization among a sample of undocumented economic migrants in Geneva, Switzerland. For the 12-months period prior to the first data collection, these migrants reported significantly fewer medical consultations than the general population and at this stage, migrants in the regularized group did not differ in their healthcare utilization from those in the control group. However, while the number of visits increased after regularization among the former, it remained stable over the two data collections among the latter. This increase suggested a positive impact of regularization on healthcare utilization, as it led to significant cross-sectional differences between regularized and control participants, even after adjusting for predisposing, enabling and health needs factors. At the within-individual level, the first-difference panel model provided further evidence of a positive impact of regularization on healthcare utilization.

In Switzerland, undocumented migrants' main reasons for avoiding healthcare utilization do not differ from those cited in other European countries (29, 36, 45). Furthermore, factors associated with healthcare utilization among this population are similar to those found in the general population in Switzerland (46). Specifically, we found that women were more likely than men to visit a doctor and that having access to a GP was a key predictor of undocumented migrants' healthcare utilization. We also found that a lower equivalent disposable income was associated with higher medical visit counts, a result consistent with previous studies about undocumented migrants' health needs in Switzerland and elsewhere, which showed that the lack of financial resources increased the odds of reporting poor health (26, 47–49). While financial barriers might hinder healthcare utilization in times of need, especially in countries where the healthcare system is predominantly market-driven, the dedicated unit for undocumented population in Geneva might contribute to bridging the gap between migrants' health needs and their healthcare access, providing a contextual explanation for the negative relationship between income and healthcare utilization. Still, we found that compared to legal residents and natives in Geneva, undocumented migrants reported fewer medical consultations despite the availability of dedicated public health services. While we could not adjust for other factors nor provide a detailed picture of the type of healthcare sought by undocumented migrants, these results are in line with previous findings in various geographical settings. Studies led in England, Denmark, Portugal, Belgium or the Netherlands consistently showed that undocumented migrants were not as likely as the legal immigrants or the natives to consult in primary care services (8, 16, 50–52). In Germany, Castañeda found that undocumented migrants tended to avoid or delay consultations in specialist care services (53). De Jonge et al. made similar observations in the Netherlands, where they found that undocumented pregnant women attended fewer pre-natal visits than their documented counterparts, a finding in line with a similar study conducted in Geneva, Switzerland (54). Overall, our results support the hypothesis that regardless of the country of residence, undocumented migrants are more likely to be disadvantaged in the utilization of healthcare services (12).

In light of this consistent association between lack of residence status and limited use of healthcare services, we hypothesized that regularization could enhance migrants' healthcare utilization through a more secured residence status. In Europe, calls for effective policies and practices improving access to healthcare for undocumented migrants have multiplied in recent years (31, 32). Yet, policy recommendations issued so far mainly focused on the organization of the healthcare system. They rarely encompassed reforms in other areas, such as in migratory or labor policies, to promote healthcare for undocumented migrants (32). To our knowledge, Belgium is the only European country in which the provision of a residence status was explicitly suggested by a panel of experts as a policy instrument to facilitate undocumented migrants' access to treatment for specific infectious diseases, such as tuberculosis (32). To date, only qualitative interviews with regularized migrants suggested that positive effects of regularization policies encompassed improved access to various public benefits such as welfare, social insurances and healthcare (55). Using a quantitative approach, this paper thus bridges a gap. It supports that policies aiming at granting undocumented migrants residence authorizations might improve healthcare utilization for this population and, as a result, foster better health in this community. It also strengthens previous findings in other contexts such as in the US, where policies promoting undocumented migrants' social integration were found to have positive effects on their healthcare utilization (33, 34).

Several limitations should be considered when interpreting the results of this study. Overall, the different sizes of our residence status groups [Regularized (*N* = 68) and Controls (*N* = 241)] reduced statistical power and increased the margins of error. Yet, despite the increased margins of error, we still found a significant association between residence status regularization and healthcare utilization, not only at the between-individual level, but also at the within-individual one. This suggests a strong effect size, i.e., a strong relationship between regularization and healthcare utilization. Nonetheless, our sampled population may not be representative of the undocumented population in Geneva and, a fortiori, in Europe due to convenience sampling. More specifically, we explored the situation of a specific group of stable, well-established undocumented workers, whose socio-economic conditions and health needs are widely different from those of newly arrived migrants at the borders of Europe (56). Furthermore, since 15% of our participants were recruited in the HUG waiting rooms, the sample might have been biased toward healthcare users, leading to slight overestimation of undocumented migrants' healthcare utilization. Convenience sampling also hampers the generalizability of our results, since we cannot exclude unobserved residual confounding due to selection bias. However, the availability of longitudinal data on hard-to-reach migrants and the use of the first-difference estimator minimizes this risk of confounding to unobserved time-variant features. Still, the results should be subject to cautious interpretation regarding causality and the underlying mechanisms at stake. Of note, we could not assess whether the effect of the residence status per se was mediated by affiliation to a health insurance due to collinearity issues. More research is thus needed to better understand the mechanisms through which regularization improves undocumented migrants' use of healthcare services and the mid-to-long term impact of this better access on migrants' health.

## Conclusion

This study supports the hypothesis that public policies aiming at granting undocumented migrants residence authorizations improve healthcare utilization for this population. It strengthens previous findings that highlighted the positive effects of public policies promoting migrants' inclusion on their use of healthcare services. More research is needed to understand the mechanisms through which regularization improves undocumented migrants' use of healthcare services.

## Data Availability Statement

The datasets presented in this article are not readily available because the datasets generated and/or analyzed during the current study are not publicly available due to the temporary embargo on data dissemination until 2023 required by the main funding agency of the study (Swiss National Fund for Scientific Research) but are available from the corresponding author on reasonable request. Requests to access the datasets should be directed to julien.fakhoury@unige.ch.

## Ethics Statement

The studies involving human participants were reviewed and approved by the Ethics Committee of Geneva Canton, Switzerland (CCER 2017–00897). The patients/participants provided their written informed consent to participate in this study.

## Author Contributions

YJ and CB-J designed the Parchemins study, the framework within which this article was elaborated. Data were collected by JF, AD, and LC. JF conducted all the analyses and drafted the manuscript. YJ, CB-J, AD, and LC proofread the manuscript. All authors approved the manuscript.

## Funding

The Parchemins study was supported by the Geneva University School of Medicine, Fondation Safra, Geneva Directorate of Health, Geneva Directorate of Social Affairs, Swiss Federal Office of Public Health, the NCCR LIVES Project and the Swiss National Fund for Scientific Research (Grant 100017_182208). Funders had no role in the development of the study design, data collection, interpretation, and dissemination.

## Conflict of Interest

The authors declare that the research was conducted in the absence of any commercial or financial relationships that could be construed as a potential conflict of interest.

## Publisher's Note

All claims expressed in this article are solely those of the authors and do not necessarily represent those of their affiliated organizations, or those of the publisher, the editors and the reviewers. Any product that may be evaluated in this article, or claim that may be made by its manufacturer, is not guaranteed or endorsed by the publisher.

## Abbreviations

95% CI: 95% confidence interval
aβ: adjusted beta coefficient
aIRR: adjusted incidence risk ratio
aOR: adjusted odds ratio
CHF: Swiss Franc
€: Euro
HUG: Geneva University Hospital
IRR: incidence risk ratio
OR: odds ratio
NGO's: non-governmental associations
SD: standard deviation
SHS: Swiss Health Survey
USD: Dollar US
UHC: Universal health coverage.
